# SAWGAN-BDCMA: A Self-Attention Wasserstein GAN and Bidirectional Cross-Modal Attention Framework for Multimodal Emotion Recognition

**DOI:** 10.3390/s26020582

**Published:** 2026-01-15

**Authors:** Ning Zhang, Shiwei Su, Haozhe Zhang, Hantong Yang, Runfang Hao, Kun Yang

**Affiliations:** 1 College of Electronic Information Engineering, Taiyuan University of Technology, Taiyuan 030024, China; 2023521567@link.tyut.edu.cn; 2 Shanxi Key Laboratory of Artificial Intelligence & Micro Nano Sensors, College of Integrated Circuits, Taiyuan University of Technology, Taiyuan 030024, China; 2024511128@link.tyut.edu.cn (S.S.); zhanghaozhe1405@link.tyut.edu.cn (H.Z.); 2023001176@link.tyut.edu.cn (H.Y.); 3 Suzhou Yakun Zhichuang Technology Co., Ltd., Suzhou 215001, China; 4 Jinan Shengquan Group Share-Holding Co., Ltd., Jinan 250000, China

**Keywords:** fine-grained emotion recognition, electroencephalography, photoplethysmography, data augmentation, cross-modal attention, multimodal signal fusion

## Abstract

Emotion recognition from physiological signals is pivotal for advancing human–computer interaction, yet unimodal pipelines frequently underperform due to limited information, constrained data diversity, and suboptimal cross-modal fusion. Addressing these limitations, the Self-Attention Wasserstein Generative Adversarial Network with Bidirectional Cross-Modal Attention (SAWGAN-BDCMA) framework is proposed. This framework reorganizes the learning process around three complementary components: (1) a Self-Attention Wasserstein GAN (SAWGAN) that synthesizes high-quality Electroencephalography (EEG) and Photoplethysmography (PPG) to expand diversity and alleviate distributional imbalance; (2) a dual-branch architecture that distills discriminative spatiotemporal representations within each modality; and (3) a Bidirectional Cross-Modal Attention (BDCMA) mechanism that enables deep two-way interaction and adaptive weighting for robust fusion. Evaluated on the DEAP and ECSMP datasets, SAWGAN-BDCMA significantly outperforms multiple contemporary methods, achieving 94.25% accuracy for binary and 87.93% for quaternary classification on DEAP. Furthermore, it attains 97.49% accuracy for six-class emotion recognition on the ECSMP dataset. Compared with state-of-the-art multimodal approaches, the proposed framework achieves an accuracy improvement ranging from 0.57% to 14.01% across various tasks. These findings offer a robust solution to the long-standing challenges of data scarcity and modal imbalance, providing a profound theoretical and technical foundation for fine-grained emotion recognition and intelligent human–computer collaboration.

## 1. Introduction

With the rapid advancement of artificial intelligence, emotion recognition has emerged as a cornerstone of human–computer interaction (HCI), enabling machines to perceive human affective states and facilitate naturalistic responses [1]. While facial expressions [2] and speech signals [3] are widely utilized in current research, they are susceptible to subjective manipulation and deliberate masking. In contrast, physiological signals are directly regulated by the Autonomic Nervous System (ANS) and peripheral nervous system (PNS), offering an objective and authentic reflection of an individual’s psychophysiological state [4]. Furthermore, the evolution of wearable technology has enabled the real-time capture of subtle physiological variations, significantly propelling research into physiology-based emotion recognition [5].

To empower machines with the capability to precisely infer human intentions, a scientific emotional definition model is fundamental. Although discrete models enumerate a limited set of basic emotions, continuous dimensional approaches have gained prominence in affective computing for their ability to refine emotional states and capture nuanced affective dynamics. The most prevalent framework is Russell’s Valence–Arousal (V-A) model, which maps emotions onto a two-dimensional plane where valence represents the degree of positivity and arousal signifies intensity. This model provides a quantitative foundation for fine-grained multi-class emotion representation [6].

Commonly used signals include electroencephalography (EEG), electrocardiography (ECG), galvanic skin response (GSR), photoplethysmography (PPG), and electromyography (EMG) [7]. Due to their non-invasive nature, EEG and PPG signals have been extensively utilized in emotion recognition tasks. As a direct modality for capturing cerebral electrical activity, EEG provides profound insights into cognitive and emotional states. PPG signals reflect cardiovascular activities regulated by the ANS, offering critical complementary information from a PNS perspective. Recent studies have demonstrated the efficacy of these modalities: Lu et al. [8] proposed a Convolution–Multilayer Perceptron Network (CMLP-Net) that effectively captures shared spatiotemporal features across EEG signal windows, achieving a 98.65% valence classification accuracy on the DEAP dataset. Qiao et al. [9] developed a Generative Adversarial Network (GAN) integrated with an attention mechanism to augment brain cognitive maps, thereby enhancing recognition accuracy to 94.87% for quaternary classification on the SEED dataset. Furthermore, Han et al. [10] introduced an Emotional Multi-scale Convolutional Neural Network (EMCNN) that enriches metadata by integrating both time- and frequency-domain information from PPG signals, attaining a quaternary accuracy of 86.1% on the DEAP dataset.

However, given the inherent complexity of human affect, the information capacity of unimodal signals remains limited, making it challenging to comprehensively capture the nuanced variations of emotional states. Integrating diverse physiological modalities can enhance information complementarity and improve recognition robustness. Consequently, deep learning-based multimodal fusion has become the mainstream paradigm.

In contemporary research, various advanced frameworks have been proposed to address multimodal challenges. For instance, the DAIL model, introduced by Li et al. [11], utilizes domain adaptation techniques to account for inter-subject variability, when peripheral physiological signals from the DEAP dataset were employed for feature extraction, classification accuracies of 86.6% for valence and 84.4% for arousal were reported. Wang et al. [12] introduced Husformer, an end-to-end multimodal Transformer designed to learn interactive representations across four modalities (EEG, EMG, GSR and eye-blink) via cross-modal mechanisms, surpassing 90% accuracy in binary classification tasks. Furthermore, the modified Cross Modal Transformer (MCMT) model, developed by Li et al. [13], addresses the issue of cross-modal inconsistency by designating EEG as the primary modality supplemented by various auxiliary signals, reaching an overall recognition precision of 92.88% on the DEAP dataset.

Despite these advancements in binary or ternary classification tasks, existing models often struggle to effectively address the challenges of fine-grained emotion recognition. The research bottlenecks are twofold: the inherent class imbalance and data scarcity in physiological datasets limit the generalization of models in high-dimensional classification, and there is an insufficient exchange of deep-seated features between EEG and PNS signals during the fusion process.

To overcome these challenges, a novel multimodal emotion recognition framework, namely the Self-Attention Wasserstein Generative Adversarial Network with Bidirectional Cross-Modal Attention (SAWGAN-BDCMA), is introduced in the present study. The primary contributions of this work are as follows:

- To expand the dataset scale, a Self-Attention Wasserstein GAN (SAWGAN) is introduced to synthesize high-quality samples through an adaptive weighting strategy and a signal smoothing mechanism, effectively alleviating the class imbalance problem.
- To facilitate high-efficiency feature representation, a dual-branch architecture is employed to process heterogeneous physiological signals independently, fully mining intra-modal spatiotemporal representations.
- To optimize the integration of deep physiological features, a Bidirectional Cross-Modal Attention (BDCMA) module is proposed to adaptively assign importance weights across modalities, enhancing information complementarity and fusion efficiency.
- The classification performance of SAWGAN-BDCMA was validated using the DEAP and ECSMP datasets, achieving an improvement of up to 14.01% over state-of-the-art (SOTA) multimodal methods.

The remainder of this paper is organized as follows: Section 2 details the architectural design of the proposed SAWGAN-BDCMA framework and the underlying principles of its constitutive modules. Section 3 presents the experimental configuration and performance evaluations conducted on the DEAP and ECSMP datasets. Section 4 provides an in-depth discussion of the unimodal testing results and the analysis of cross-modal fusion weights. Finally, Section 5 concludes the paper and outlines potential directions for future research.

## 2. Materials and Methods

**Figure 1:**
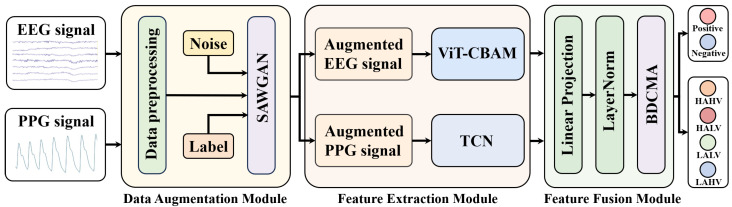
The framework of the proposed multimodal emotion recognition model SAWGAN-BDCMA.

This section elaborates on the modules and methods employed in the proposed multimodal emotion recognition model SAWGAN-BDCMA, including the data augmentation approach, the dual-branch feature extraction model, and the feature fusion method, as illustrated in Figure 1.

### 2.1. Data Augmentation Module

The limited size of EEG and PPG datasets, coupled with class imbalance, hampers the training of robust emotion recognition models. To curb overfitting and improve generalization, this study expands the training set via data augmentation. Given the high dimensionality and complexity of physiological signals, a generative augmentation strategy based on Wasserstein Generative Adversarial Networks (WGAN) is adopted. Compared to classical GANs that rely on Jensen-Shannon divergence, WGAN is selected for its ability to provide a smoother optimization landscape via Wasserstein distance, which effectively mitigates training instability and mode collapse—common challenges when modeling high-dimensional physiological distributions [14,15].

#### 2.1.1. Data Preprocessing

Prior to data augmentation, raw EEG and PPG signals undergo necessary preprocessing, including baseline removal, denoising and normalization to ensure numerical stability during training. Normalization is performed by subtracting the mean and dividing by the standard deviation for each channel. Subsequently, the data are clipped to the range [−3, 3] following the 3σ rule, which effectively eliminates potential outliers (representing less than 0.3% of the distribution) and prevents gradient instability caused by extreme values. Finally, the data are linearly rescaled to the interval [−1, 1]. This rescaling not only preserves the signal morphology within a bounded symmetric range but also aligns with the bounded output of the generator’s Tanh activation function, thereby preventing saturation and facilitating faster convergence during the adversarial game. After preprocessing, the data are reshaped into the format (N×C×T), where $N=N_{\mathrm{trial}}\times N_{\mathrm{segment}}$ denotes the total number of samples (trials × segments), *C* represents the number of channels (32 for DEAP and 7 for ECSMP in EEG; 1 for PPG), and *T* is fixed at 256 time steps.

#### 2.1.2. SAWGAN Architecture

**Figure 2:**
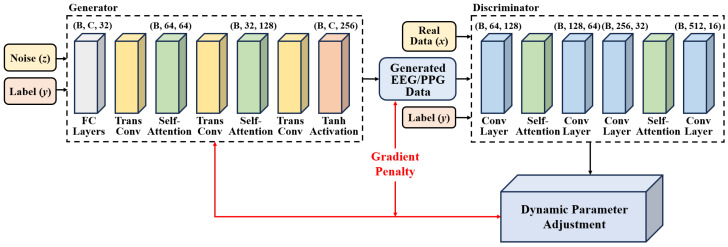
The specific structure and principles of SAWGAN.

Expanding upon the WGAN structure, the proposed SAWGAN incorporates two critical enhancements to handle heterogeneous physiological signals. First, it adopts the Conditional WGAN with Gradient Penalty (CWGAN-GP) as its foundation, leveraging class labels to supervise the synthesis process of label-specific samples [16]. Second, to tackle the inherent physiological variances between EEG and PPG, where EEG reflects multi-channel cerebral dynamics and PPG captures single-channel cardiovascular activity, a self-attention mechanism is integrated into both the generator and discriminator. This allows the model to adaptively capture modality-specific features: cross-channel spatial-temporal correlations for EEG and morphological periodicities for PPG. The specific architecture of SAWGAN is illustrated in Figure 2.

The specific arrangement of SAWGAN components is tailored to accommodate the complex properties of physiological data. Specifically, self-attention modules are strategically integrated after convolutional layers to compensate for the limited receptive field of standard filters, enabling the model to capture long-range temporal dependencies and global cross-channel correlations essential for accurate EEG and PPG synthesis. Furthermore, the three-layer fully connected network acts as a high-dimensional feature mapper to effectively project the noise-label latent space into a structured initial representation.

The generator $G_{\theta }$ takes a noise vector $z\in R^{d}$ and the corresponding class label y as inputs. The label is processed through an embedding layer and concatenated with z. The concatenated vector is transformed by a three-layer fully connected network and reshaped into an initial low-resolution representation of dimensions (*B*, *C*, 32), where *B* is the batch size and *C* denotes the number of channels. Subsequently, transposed convolutional layers upsample the feature map from (*B*, *C*, 32) to (*B*, 64, 64), then to (*B*, 32, 128), and finally to the target output shape (*B*, *C*, *T*), where T=256 and *C* conforms to the target signal channels. Self-attention blocks are inserted after the first and third transposed convolutional layers to capture long-range dependencies. The generator’s output is mapped to the range [−1, 1] using a Tanh activation:

(1) $$G_{\theta }\left(z,y\right)=\tanh\left(\mathrm{UpConv}\left(\mathrm{FC}\left(\left[z,\mathrm{Embed}\left(y\right)\right]\right)\right)\right)$$

where z is the random noise vector and y denotes the conditional label.

The discriminator $D_{\phi }$ receives either real physiological signal segments $x\in R^{B\times C\times T}$ or generated pseudo segments $x_{p}=G_{\theta }\left(z,y\right)$, along with their labels y, where C=32 or 7 for EEG or C=1 for PPG, and T=256. The label is embedded, spatially expanded, and incorporated into the input feature map via broadcasting. As depicted in Figure 2, a stack of temporal convolutional blocks reduces the temporal resolution while increasing channel dimensionality. Subsequently, self-attention modules and global average pooling (GAP) are applied to extract a feature vector of size (B,512), which is passed through a linear classifier to produce the output $D_{\phi }\left(x,y\right)$:

(2) $$D_{\phi }\left(x,y\right)=\mathrm{Linear}\left(\mathrm{GAP}\left(\mathrm{SA}\left(\mathrm{Conv}\left(x+\alpha \cdot \mathrm{Embed}\left(y\right)\right)\right)\right)\right)$$

where ϕ represents the network parameters of the discriminator, Embed(y) denotes the label embedding, and α is a learnable weight that adaptively modulates the influence of the conditional information.

In this paper, the Wasserstein distance with gradient penalty is employed as the training criterion. The loss function of the discriminator $L_{D}$ is defined as:

(3) $$L_{D}=-E_{x∼p_{\mathrm{real}}}\left[D_{\phi }\left(x,y\right)\right]+E_{x_{p}∼p_{\mathrm{pseudo}}}\left[D_{\phi }\left(x_{p},y\right)\right]+\lambda_{\mathrm{gp}}L_{\mathrm{GP}}$$

where $L_{\mathrm{GP}}$ is the gradient penalty to enforce the Lipschitz constraint:

(4) $$L_{\mathrm{GP}}=E_{\hat{x}∼p_{\hat{x}}}[(∥\nabla_{\hat{x}}D_{\phi }\left(\hat{x},y\right){∥}_{2}-1{)}^{2}]$$

where $\hat{x}$ is a random interpolation between real samples x and pseudo samples $x_{p}$.

SAWGAN introduces a dynamic optimization strategy compared to conventional Wasserstein GAN with Gradient Penalty (WGAN-GP) [17]. The generator $G_{\theta }$ is trained to minimize the combined objective $L_{G}$, which facilitates label-guided manifold alignment through class-conditional variables y:

(5) $$L_{G}=\alpha_{t}L_{\mathrm{adv}}+\beta_{t}L_{\mathrm{align}}+\gamma_{t}L_{\mathrm{entropy}}$$

where $L_{\mathrm{adv}}$ is the adversarial loss defined as: $L_{\mathrm{adv}}=-E_{z∼p_{z}}\left[D_{\phi }\left(G_{\theta }\left(z,y\right),y\right)\right]$.

Unlike standard WGANs that rely on static hyperparameters, SAWGAN reformulates the parameter update rule as:

(6) $$\theta_{t+1}=\theta_{t}-\eta_{G}^{\left(t\right)}\nabla_{\theta }L_{G}$$

where the learning rate $\eta_{G}^{\left(t\right)}$ and the objective coefficients $\left(\alpha_{t},\beta_{t},\gamma_{t}\right)$ are time-varying parameters that evolve adaptively based on the competitive dynamics between $G_{\theta }$ and $D_{\phi }$.

Specifically, $L_{\mathrm{align}}$ is the manifold alignment loss, which ensures that the synthetic samples preserve the underlying topological structure of the real physiological distribution for each emotion class. $L_{\mathrm{entropy}}$ represents the information entropy regularizer, introduced to maximize the diversity of generated samples and prevent the mode collapse common in high-dimensional physiological signal synthesis. The overall optimization process is detailed in Algorithm 1.
**Algorithm 1** Self-Adaptive Parameter Adjustment in SAWGAN**Input:** 
Generator $G_{\theta }$, Discriminator $D_{\phi }$, real data x, noise z∼p(z), adaptation rate δ, initial weights $\left(\alpha_{0},\beta_{0},\gamma_{0}\right)$**Output:** 
Optimized parameters θ,ϕ, and adaptive hyper-parameter trajectories  1:**for** each iteration *t* **do**  2:   Generate pseudo data $x_{p}=G_{\theta }\left(z,y\right)$  3:   Compute discriminator loss: $L_{D}=-E\left[D_{\phi }\left(x,y\right)\right]+E\left[D_{\phi }\left(x_{p},y\right)\right]+\lambda_{\mathrm{gp}}L_{\mathrm{GP}}$  4:   Compute generator loss: $L_{G}=\alpha_{t}L_{\mathrm{adv}}+\beta_{t}L_{\mathrm{align}}+\gamma_{t}L_{\mathrm{entropy}}$  5:   Update weights adaptively: $\alpha_{t+1},\beta_{t+1},\gamma_{t+1}\leftarrow \mathrm{Normalize}\left(\alpha_{t}+\delta \nabla L_{G}\right)$  6:   Adjust learning rates: $\eta_{G}^{\left(t+1\right)},\eta_{D}^{\left(t+1\right)}\leftarrow \eta_{G}^{\left(t\right)}\left(1+\delta \;\mathrm{sign}\left({\dot{L}}_{G}\right)\right),\;\eta_{D}^{\left(t\right)}\left(1-\delta \;\mathrm{sign}\left({\dot{L}}_{D}\right)\right)$  7:   Update parameters: $\theta_{t+1}\leftarrow \theta_{t}-\eta_{G}^{\left(t\right)}\nabla_{\theta }L_{G}$, $\phi_{t+1}\leftarrow \phi_{t}-\eta_{D}^{\left(t\right)}\nabla_{\phi }L_{D}$  8:**end for**  9:**return** Optimized parameters θ and ϕ

As specified in the algorithm’s output, the adaptive module yields a set of optimized parameters (θ,ϕ) that have converged under a balanced adversarial game. The adaptation involves the rate δ and the loss change rate $\dot{L}$, which direct the trajectory of hyper-parameter updates. By dynamically modulating learning rates (LR) and loss coefficients according to real-time gradient statistics, the controller ensures that the model bypasses suboptimal local minima and mitigates the risk of mode collapse, ultimately producing high-fidelity synthetic physiological samples.

### 2.2. Feature Extraction Module

**Figure 3:**
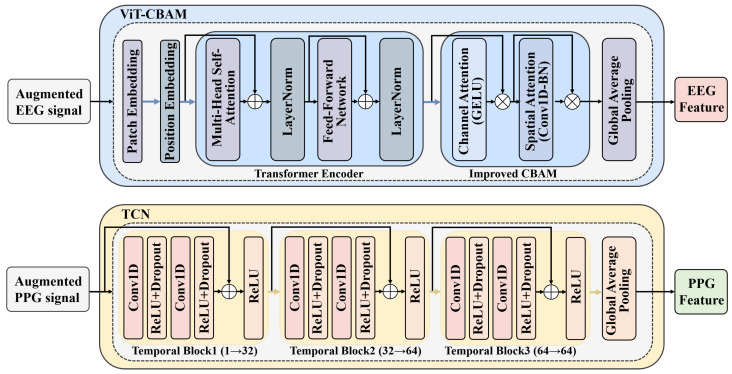
Visualisation of the dual-branch feature extraction model structure.

This section describes the signal feature extraction module in SAWGAN-BDCMA, the specific architecture of which is illustrated in Figure 3. As shown, this module is composed of a ViT-CBAM branch for processing EEG signals and a TCN branch for handling PPG signals.

#### 2.2.1. ViT-CBAM

The EEG branch adopts a Vision Transformer (ViT) backbone [18] augmented with an enhanced Convolutional Block Attention Module (CBAM) [19] for spatiotemporal modeling of multi-channel signals. Input segments are first tokenized by a Patch Embedding module, then processed by a Transformer encoder to capture long-range temporal dependencies and inter-channel relations. Unlike conventional CBAM, which modulates purely convolutional features, the proposed variant is placed after the Transformer encoder to reweight channel and pseudo-spatial dimensions of the global ViT features. This placement allows attention to operate on richer, context-integrated representations, strengthening discrimination of emotion-relevant activity patterns. The overall architecture consists of Patch Embedding, Transformer Encoder, and the post-encoder CBAM attention block (see Figure 3).

- Patch Embedding Module: EEG signals exhibit a structured organization across channels and time, motivating a decoupled spatiotemporal encoding strategy during patch embedding. The input is first processed by a temporal convolutional layer Conv2d(1, 24, (1, 25)), which operates along the time dimension to capture dynamic patterns within short-term intervals. Subsequently, spatial convolution (Conv2d(24, 24, (32, 1))) is applied to integrate information across brain regions in the channel dimension, enabling the encoding of inter-channel coupling relationships across 32 electrode channels. Thereafter, dimensionality reduction and feature rearrangement are achieved through pooling and a 1×1 convolutional projection, mapping features into the token representation space: $Z\in R^{B\times P\times D}$.
- Transformer Encoder: This module is designed to learn global dependencies in EEG signals. Each patch sequence, augmented with a learnable classification (CLS) token and positional encoding to preserve temporal information and enable global representation, is fed into a multi-layer Transformer encoder. Within each encoder layer, the features are updated through Multi-Head Self-Attention (MHSA) mechanisms and a Feed-Forward Network (FFN). The feature update process can be formulated as follows:
  (7) $$\mathrm{Attention}\left(Q,K,V\right)=\mathrm{Softmax}\left(\frac{QK^{⊤}}{\sqrt{d_{k}}}\right)V$$
  (8) $$Z_{l+1}=\mathrm{LayerNorm}\left(Z_{l}+\mathrm{Attention}\left(Z_{l}\right)\right)$$
  (9) $$Z_{l+1}=\mathrm{LayerNorm}\left(Z_{l+1}+\mathrm{FFN}\left(Z_{l+1}\right)\right)$$
  where Q, K, and V represent the Query, Key, and Value matrices, respectively, and $d_{k}$ denotes the attention dimension. The FFN consists of a two-layer feed-forward network with GELU activation. Compared to ReLU, GELU’s smoother nonlinearity is more effective at representing the complex neural oscillations inherent in EEG data. The MHSA enables the modeling of interdependencies across different channels and temporal segments, while residual connections and LayerNorm ensure stable training of deep architectures.
- Improved CBAM: The improved CBAM is composed of Channel Attention (CA) and Spatial Attention (SA). In the CA stage, global descriptors are extracted from each channel using parallel average pooling and max pooling. The pooled descriptors undergo a two-layer mapping where GELU replaces ReLU to provide smoother nonlinearities, enhancing inter-channel modeling and feature stability. The outputs of the average-pooled and max-pooled branches are summed and passed through a sigmoid, yielding channel-wise attention weights that adaptively emphasize informative EEG channels and suppress less relevant activity. This mechanism improves modeling of inter-channel importance while preserving feature stability before subsequent spatial recalibration. The process can be expressed as follows:
  (10) $$X^{\prime }=X⊙\sigma \left(\mathrm{FC}(\mathrm{GELU}(\mathrm{FC}(\mathrm{AvgPool}\left(X\right)\right)))+\mathrm{FC}(\mathrm{GELU}(\mathrm{FC}(\mathrm{MaxPool}\left(X\right)))))$$
  where ⊙ denotes the element-wise multiplication operation.

In the SA module, average pooling and max pooling are applied to the feature $X^{\prime }$, and the results are subsequently concatenated. A one-dimensional convolution is then employed to capture dependencies across different positions in the sequence. Following the convolution operation, a Batch Normalization (BN) layer is introduced to stabilize the feature distribution. Additionally, an adjustable convolution kernel size *k* is incorporated to extract temporal features at different scales, enabling the model to more flexibly focus on spatial patterns in EEG signals. The final attention-enhanced features are expressed as:

(11) $$X^{\prime \prime }=X^{\prime }⊙\sigma \left(\mathrm{BN}\left(\mathrm{Conv}1D\left(\left[F_{\mathrm{avg}}^{s};F_{\max}^{s}\right]\right)\right)\right)$$

where $F_{\mathrm{avg}}^{s}$ and $F_{\max}^{s}$ are the features obtained by applying average pooling and max pooling to $X^{\prime }$, respectively. Finally, the GAP is further employed to derive the EEG branch feature representation: $x_{\mathrm{EEG}}=\mathrm{GAP}\left(X^{\prime \prime }\right)$.

#### 2.2.2. TCN

The PPG branch employs a Temporal Convolutional Network (TCN) [20] to extract temporal dependencies from PPG waveforms. By integrating causal convolutions, dilated convolutions, and residual connections, the network effectively captures both short-term and long-term temporal dynamics of the signal.

Given an input sequence $X\in R^{B\times 1\times T}$, the dilated convolution operation in each temporal block is defined as:

(12) $$H\left(t\right)=\sum_{i=0}^{k-1}w_{i}\cdot X_{t-d\cdot i}$$

where k is the kernel size and d is the dilation rate, which expands exponentially (d∈{1,2,4}) across successive blocks. Since the effective receptive field of the network increases with dilation, the model can perceive long-range temporal dependencies without losing resolution. Each block output is computed as:

(13) $$Y=\mathrm{ReLU}(F\left(X\right)+W_{r}X)$$

where F(X) denotes the transformation of two dilated convolutions with nonlinear activations, and $W_{r}$ represents the residual projection ensuring channel consistency. This residual mechanism stabilizes training and prevents gradient vanishing.

After three temporal blocks with channel widths [32, 64, 64], the final feature map $H_{3}$ undergoes the GAP along the temporal dimension to obtain a compact feature representation:

(14) $$x_{\mathrm{PPG}}=\frac{1}{T^{\prime }}\sum_{t=1}^{T^{\prime }}H_{3}\left[:,:,t\right]$$

### 2.3. Feature Fusion Module

**Figure 4:**
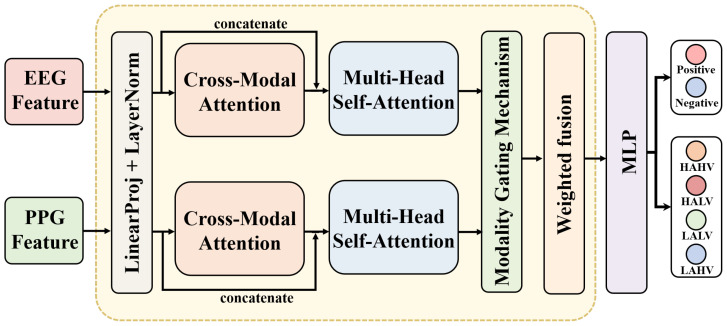
Visualisation of the BDCMA multimodal feature fusion mechanism architecture.

Following EEG and PPG feature extraction, a BDCMA is used for deep interaction and adaptive fusion of signal features. The overall structure of the proposed model is illustrated in Figure 4. Unlike unidirectional fusion methods, the bidirectional mechanism ensures that the deep spatiotemporal features of EEG and the morphological patterns of PPG can mutually guide each other’s recalibration. Specifically, the cross-modal attention maps are designed to calculate the correlation matrix between modalities, allowing the model to adaptively suppress redundant noise in one modality while emphasizing complementary emotional cues in the other. This symmetrical design minimizes information loss during the fusion process, ensuring that the final joint representation is both discriminative and robust against modality-specific noise.

Initially, the EEG and PPG features $x_{\mathrm{EEG}}\in R^{d_{\mathrm{eeg}}}$ and $x_{\mathrm{PPG}}\in R^{d_{\mathrm{ppg}}}$, obtained from the feature extraction network, are mapped to a unified representation space. Through linear projection and layer normalization, they are projected into n feature tokens, denoted as $Z_{\mathrm{EEG}}\in R^{n\times d},Z_{\mathrm{PPG}}\in R^{n\times d}$. These features are then fed into the BDCMA module, where the EEG features serve as Key-Value pairs and the PPG features act as the Query for one attention interaction. Simultaneously, the PPG features serve as Key-Value pairs and the EEG features act as the Query for the reverse interaction. This yields: ${\tilde{Z}}_{\mathrm{EEG}}=\mathrm{CrossAttn}\left(Z_{\mathrm{EEG}},Z_{\mathrm{PPG}},Z_{\mathrm{PPG}}\right),{\tilde{Z}}_{\mathrm{PPG}}=\mathrm{CrossAttn}\left(Z_{\mathrm{PPG}},Z_{\mathrm{EEG}},Z_{\mathrm{EEG}}\right)$.

The original tokens are then concatenated with the cross-modal attention outputs to form an extended sequence:

(15) $$S_{\mathrm{EEG}}=\left[Z_{\mathrm{EEG}};{\tilde{Z}}_{\mathrm{PPG}}\right]$$

(16) $$S_{\mathrm{PPG}}=\left[Z_{\mathrm{PPG}};{\tilde{Z}}_{\mathrm{EEG}}\right]$$

Subsequently, MHSA is applied to the fused token sequence to capture global dependencies, integrating intra-modal structure with context transferred across modalities [21]. The resulting sequence is then pooled to yield fixed-length, modality-level representations $f_{\mathrm{EEG}}$ and $f_{\mathrm{PPG}}$.

A modality gating mechanism is introduced to adaptively determine the fusion weights between EEG and PPG at the sample level through:

(17) $$w=\mathrm{Softmax}\left(\frac{W_{g}\left[f_{\mathrm{EEG}};f_{\mathrm{PPG}}\right]}{T}\right)$$

where *T* is a temperature parameter controlling the smoothness of the weights, and $w=[w_{\mathrm{EEG}},w_{\mathrm{PPG}}]$. The final fused features are computed as:

(18) $$f_{\mathrm{fused}}=w_{\mathrm{EEG}}\cdot f_{\mathrm{EEG}}+w_{\mathrm{PPG}}\cdot f_{\mathrm{PPG}}$$

This mechanism adaptively allocates modality importance based on sample characteristics, thereby enhancing the model’s robustness in scenarios with imbalanced multimodal features.

## 3. Experiments and Results

The evaluation was performed on the DEAP and ECSMP datasets, with ablation studies designed to quantify the contribution of each component.

### 3.1. Dataset Partitioning

DEAP is a public multimodal affect dataset comprising recordings from 32 participants who viewed 40 music video clips. In the present study, EEG from the first 32 channels and PPG from channel 39 were used, each sampled at 128 Hz [22]. For each trial, the final 60 s of EEG and PPG were extracted. Labeling rules for the binary and quaternary settings are summarized in Table 1 and Table 2.

The ECSMP dataset [23] is a comprehensive multimodal repository containing data from 89 healthy college students, including EEG, ECG, PPG, Electrodermal Activity (EDA), body temperature, and accelerometer recordings. Emotional states in this dataset are categorized into six distinct labels: neutral, fear, sadness, happiness, anger, and disgust (originally indexed as 101–106, mapped to 0–5 in this study). Since simultaneous EEG and PPG recordings were not available for all participants across all emotional states, this study focuses on a specific subset of 30 participants whose data provided a complete and synchronized representation of both target modalities.

### 3.2. Training Strategy

To comprehensively evaluate the classification performance of the proposed model on unknown data, the DEAP and ECSMP datasets were subjected to 10-fold cross-validation in this study, with an 8:1:1 split for training, validation, and testing within each fold. To ensure independent verification and reproducibility, manual seeds (42) were fixed for all weight initializations and data shuffling processes. The final performance metrics were obtained by averaging the results of the 10 experiments. During the training phase, an early stopping strategy with a patience of 20 epochs was implemented based on the validation loss to prevent overfitting. Models were implemented in PyTorch 1.9.1 with CUDA 11.1 and trained on an AMD Ryzen 9 5950X CPU with an NVIDIA RTX 3090 GPU (24 GB).

The SAWGAN module employed a 128-dimensional latent space, 32 batch size, and 120 iterations. Adhering to the WGAN-GP protocol [17], initial hyperparameters were set at $n_{critic}=5$ and $\lambda_{gp}=10$ to ensure stable optimization and accurate distance approximation. Initial LRs were $1\times 10^{-4}$ for the generator and $7\times 10^{-5}$ for the discriminator. Feature extraction hyperparameters were configured as follows: the ViT-CBAM utilized a 32-pixel patch size, 3 layers, and 4 heads (50 epochs, LR $1\times 10^{-3}$), while the TCN employed 4 residual blocks with dilation factors of 1, 2, 4, 8 and a kernel size of 3 (200 epochs, LR $1\times 10^{-4}$). Finally, the fusion module was trained for 200 epochs using the AdamW optimizer (LR $1\times 10^{-4}$, weight decay $1\times 10^{-5}$) and a Weighted Cross-Entropy Loss to address class imbalance, with a global batch size of 64.

### 3.3. Experimental Results

#### 3.3.1. Results on the DEAP Dataset

To evaluate the effectiveness of the SAWGAN module, experiments were conducted on the DEAP dataset with three augmentation levels: 0.5×, 1×, and 2×, representing the proportional expansion of the original dataset. The model was trained using concatenated original and augmented data. Table 3 and Table 4 present the binary and quaternary classification performance under different augmentation levels.

As shown in Table 3 and Table 4, consistent performance improvements are observed across all evaluation metrics as the data augmentation levels increase. Without augmentation (0×), the model achieves only 76.01% ± 2.85% and 68.37 ± 3.35% accuracies for binary and quaternary classification, respectively, with correspondingly low precision, recall, and F1-score. The best performance is achieved at 2×, with accuracy values of 94.25 ± 1.36% (binary) and 87.93 ± 1.37% (quaternary). These trends indicate that the generated data effectively broadened the training distribution and that the proposed feature extraction and fusion methods are effective.

#### 3.3.2. Results on the ECSMP Dataset

To further validate the generalization capability of the SAWGAN-BDCMA framework across diverse datasets and more complex affective computing tasks, a six-class emotion recognition experiment was conducted on the ECSMP dataset. Table 5 summarizes the performance metrics across various augmentation levels.

The experimental results in Table 5 demonstrate that the SAWGAN-BDCMA framework maintains exceptional performance consistency and robustness even as task complexity increases. The baseline accuracy of 79.75 ± 3.71% (0×) culminates in a peak of 97.49 ± 1.16% (2×) following SAWGAN-based augmentation. Consistent with the trends identified in the DEAP dataset, the model’s performance exhibits a monotonic improvement as the augmentation factor increases. This sustained improvement validates the module’s capacity to synthesize high-fidelity physiological signals, thereby diversifying the training distribution and empowering the extraction of discriminative features for granular six-class emotion recognition.

#### 3.3.3. Performance Comparison with the SOTA Models

In this study, four SOTA multimodal emotion recognition methods were evaluated and benchmarked using the DEAP and ECSMP datasets, with a detailed performance analysis presented in Table 6. To ensure a rigorous and fair comparison, all baseline models were re-implemented under a unified experimental protocol. This involves utilizing an identical hardware environment, the same data preprocessing pipelines (as detailed in Section 2.1.1), and a consistent 10-fold cross-validation strategy across all tests.

As summarized in Table 6, SAWGAN-BDCMA consistently outperformed all benchmarked SOTA methods, including Husformer [12], DAIL [11], MCMT [13], and the Multimodal-Driven Fusion Data Augmentation Framework [24]. Compared to these advanced pipelines, the proposed model achieved accuracy improvements ranging from 0.57% to 14.01% across different datasets. Notably, while the performance of domain-adaptation-based models like DAIL [11] and transformer-based architectures such as Husformer [12] showed varying degrees of decline as classification complexity increased, the proposed SAWGAN-BDCMA maintained superior robustness, reaching a peak accuracy of 97.49% on the ECSMP six-class task.

### 3.4. Feature Visualization

To assess the efficacy of SAWGAN and the feature extraction module, visual analyses were conducted on the original data, the augmented data, and their derived representations of DEAP and ECSMP.

**Figure 5:**
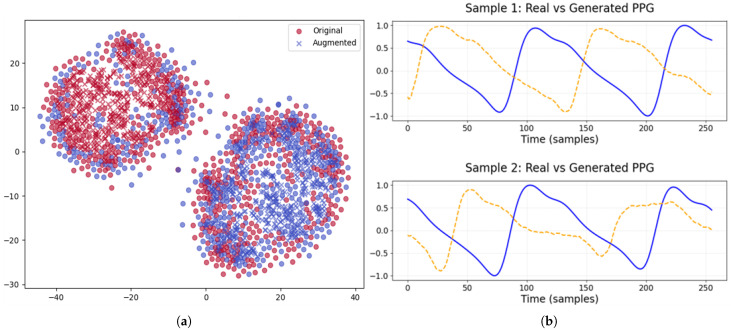
Comparison of original and augmented data on DEAP: (**a**) EEG data. Original and augmented samples are denoted by circles and crosses, with high and low valence represented in red and blue. (**b**) PPG signal waveforms. The solid blue line represents a real data sample, while the dashed yellow line corresponds to the generated sample.

**Figure 6:**
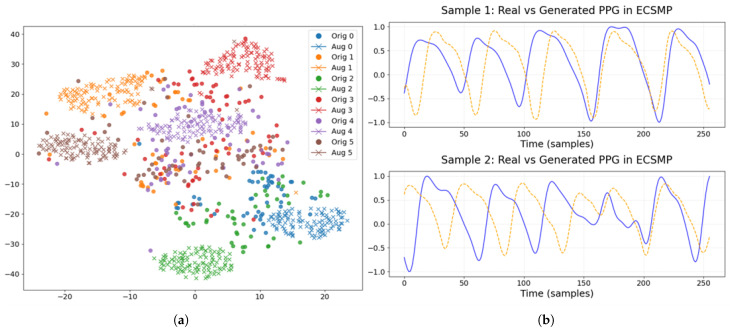
Comparison of original and augmented data on ECSMP: (**a**) EEG data. Original and augmented samples are represented by circles and crosses, respectively, with distinct colors representing different emotional states. (**b**) PPG signal waveforms. The solid blue line represents a real data sample, while the dashed yellow line corresponds to the generated sample.

As illustrated in Figure 5 and Figure 6, the original and augmented physiological signals exhibit high distributional consistency across both DEAP and ECSMP datasets. In the t-SNE visualizations of EEG data, the cluster regions of original samples (circles) and synthetic samples (crosses) show substantial overlap. This indicates that SAWGAN effectively captures the intrinsic manifold of EEG features, enhancing dataset diversity without introducing significant distribution shifts. Similarly, the augmented PPG waveforms maintain high fidelity to the original signals, preserving critical temporal characteristics such as periodic patterns and heart rate variability (HRV) features. Even on the more complex six-class ECSMP dataset, SAWGAN accurately captures the dynamic transitions of physiological signals, providing a robust data source for subsequent feature extraction.

**Figure 7:**
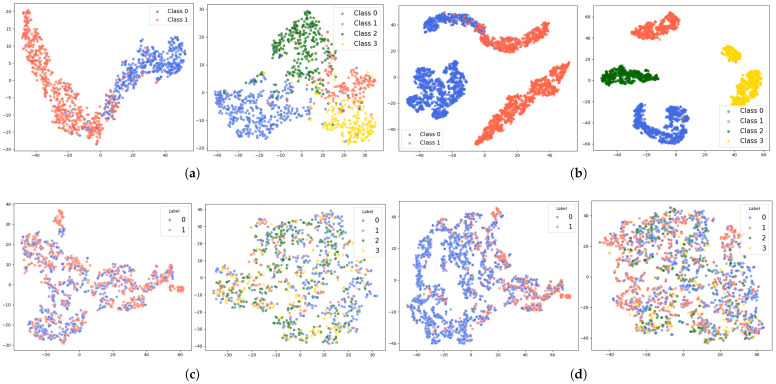
t-SNE feature visualization of original and augmented data from the DEAP dataset using the dual-branch model: (**a**) Original EEG features. (**b**) EEG features with 2× augmented data. (**c**) Original PPG features. (**d**) PPG features with 2× augmented data.

**Figure 8:**
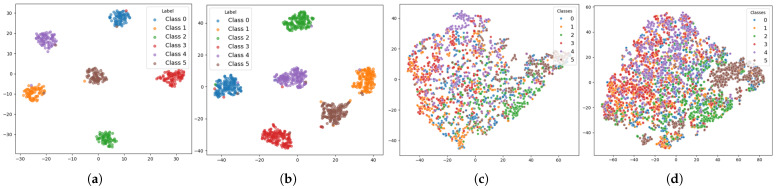
t-SNE feature visualization of original and augmented data from the ECSMP dataset using the dual-branch model: (**a**) Original EEG features. (**b**) EEG features with 2× augmented data. (**c**) Original PPG features. (**d**) PPG features with 2× augmented data.

Figure 7 and Figure 8 visually demonstrate the feature extraction performance of the dual-branch model on the DEAP and ECSMP datasets. As shown in Figure 7a and Figure 8a, the ViT-CBAM architecture successfully learns discriminative representations from the original EEG data, evidenced by the distinct clustering across categories. The introduction of 2× augmented EEG data in Figure 7b and Figure 8b yields richer internal structures and sharper inter-class boundaries, particularly within the multi-class ECSMP environment.

Regarding the PPG modality, Figure 7c and Figure 8c exhibit significant overlap among data points when utilizing only original signals, indicating the limited initial separability of the TCN model. However, incorporating 2× augmented PPG data enhances feature aggregation, as illustrated in Figure 7d and Figure 8d. The diverse sample information provided by the SAWGAN module enables both the ViT-CBAM and TCN models to capture more robust spatiotemporal dynamics, resulting in superior discriminative power even for the complex six-class classification task in the ECSMP dataset.

### 3.5. Ablation Experiments

**Figure 9:**
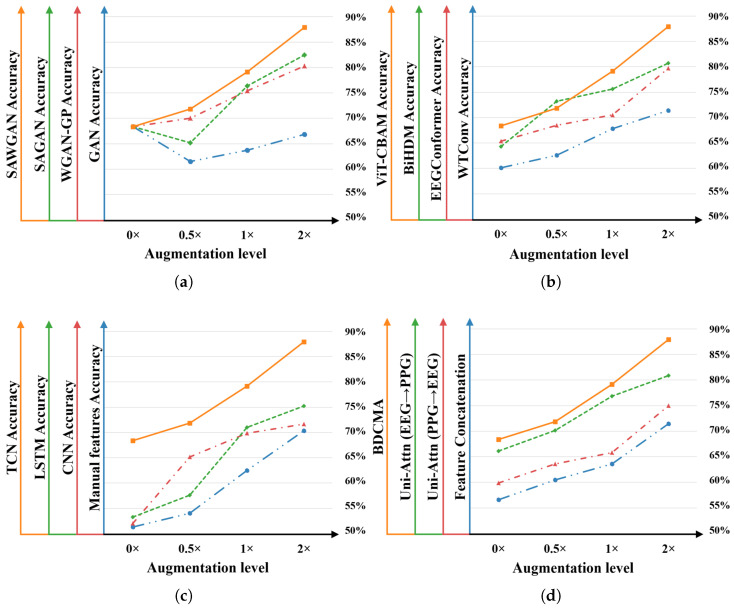
Ablation study results of individual modules for quaternary classification on the DEAP dataset: (**a**) Different data augmentation techniques. (**b**) Various EEG signal feature extraction methods. (**c**) Diverse PPG signal feature extraction approaches. (**d**) Different feature fusion strategies.

**Figure 10:**
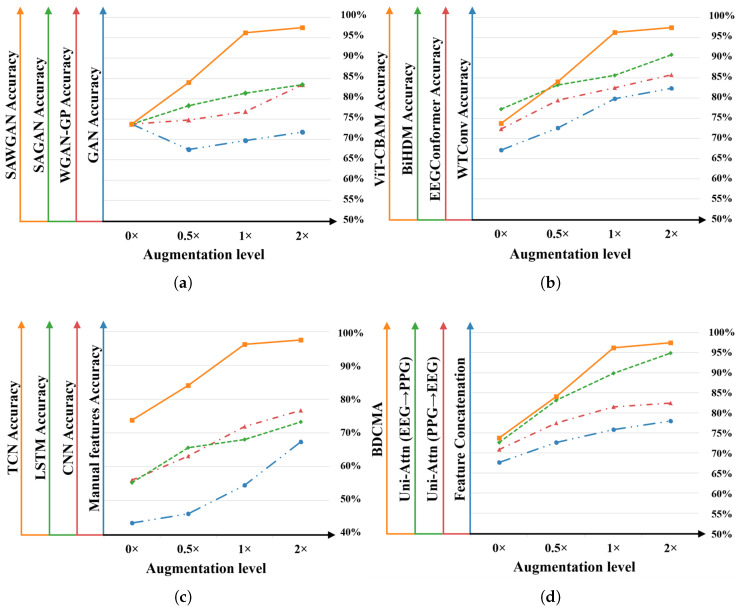
Ablation study results of individual modules for six classifications on the ECSMP dataset: (**a**) Different data augmentation techniques. (**b**) Various EEG signal feature extraction methods. (**c**) Diverse PPG signal feature extraction approaches. (**d**) Different feature fusion strategies.

Ablation studies were conducted to evaluate the performance of the proposed model and the individual contributions of its components. The corresponding experimental results are presented in Figure 9 and Figure 10.

#### 3.5.1. Ablation Study of Data Augmentation Methods

To evaluate the individual contributions of the proposed components, the self-attention mechanism and Wasserstein optimization were decoupled, with results illustrated in Figure 9a and Figure 10a. While the WGAN-GP improves training stability relative to vanilla GANs, the absence of self-attention limits its capacity to capture long-range dependencies in physiological signals, resulting in lower accuracies of 80.30% and 82.57% at 2× augmentation. Conversely, the SAWGAN integrates both mechanisms and achieves peak performance, demonstrating a powerful synergy between global feature relationship modeling and gradient-penalty-based optimization. Even at high augmentation ratios, SAWGAN exhibits stable performance gains while preserving the underlying statistical characteristics of the original multimodal signals.

#### 3.5.2. Ablation Study of Feature Extraction Modules

Various feature extraction methods were evaluated on EEG and PPG signals to assess the contribution of the dual-branch module. For EEG (Figure 9b and Figure 10b), the proposed ViT-CBAM achieved peak accuracies of 87.93% and 97.49% on the DEAP and ECSMP datasets, respectively, outperforming WTConv [25], EEGConformer [26], and BiHDM [27]. This gain is attributable to its hybrid design, which combines global context modeling via a Vision Transformer with targeted channel–spatial reweighting. Similarly, for PPG (Figure 9c and Figure 10c), the TCN-based branch outperformed manual feature engineering (70.34%) and deep learning baselines. While CNNs [28] primarily capture local structures and LSTMs [29] are susceptible to gradient degradation, the TCN leverages dilated causal convolutions to model long-range temporal dependencies, yielding superior feature extraction. These results demonstrate that the tailored dual-branch design provides a robust foundation for subsequent cross-modal fusion.

#### 3.5.3. Ablation Study of Feature Fusion Strategies

To verify the efficacy of the BDCMA mechanism, comparative experiments were conducted against three alternative strategies: feature concatenation, unidirectional attention (PPG-to-EEG), and unidirectional attention (EEG-to-PPG). As summarized in Figure 9d and Figure 10d, feature concatenation yields the lowest performance due to its inability to model complex inter-modal correlations. While unidirectional attention improves results, the EEG-to-PPG configuration consistently outperforms PPG-to-EEG, suggesting that high-dimensional spatiotemporal EEG features provide superior latent guidance for PPG recalibration. Moreover, BDCMA achieves the highest accuracy in all scenarios. Unlike unidirectional methods, BDCMA’s symmetrical mutual-feedback mechanism minimizes information loss and enhances the synergy between central and PNS signals. As the augmentation factor increases, the widening performance gap further substantiates BDCMA’s superior ability to capture discriminative emotional cues from enriched data distributions.

## 4. Discussion

To ensure high fidelity in the augmented data, a rigorous statistical and physiological evaluation of the SAWGAN-generated signals was conducted. The synthetic EEG and PPG signals exhibit minimal statistical deviation from real distributions, with mean differences of 0.0267 and 0.0112, respectively, while the low correlation structure differences confirm that inter-channel dependencies are effectively preserved. Crucially, the physiological integrity of the generated PPG was validated via HRV analysis. The mean RR interval difference was merely 0.0026 s, an error significantly lower than a single sampling period. Despite minor variances in standard deviation of normal-to-normal intervals (SDNN, 0.0247 s) and root mean square of successive differences (RMSSD, 0.0419 s), the overall physiological profile remains highly consistent with the original signals. These results substantiate that the SAWGAN module synthesizes signals that are both mathematically and physiologically valid, effectively enhancing emotion recognition performance.

Furthermore, the influence of various activation functions within the ViT-CBAM module was investigated. Comparative evaluations on the DEAP and ECSMP datasets demonstrated that models utilizing smooth, non-linear functions, such as GELU and ELU, consistently outperformed ReLU and LeakyReLU (as detailed in Table 7). Specifically, the GELU-based configuration yielded 3.67% and 1.48% performance increments over ReLU and ELU, respectively, in the DEAP quaternary task, while attaining performance parity with ELU in the ECSMP six-class task. This disparity likely stems from GELU’s superior capability in capturing the stochastic and non-linear dynamics of physiological signals like EEG. Moreover, GELU exhibits inherent architectural synergy with the Transformer backbone, facilitating the preservation of richer discriminative features during cross-modal fusion and ensuring robust recognition across complex affective states.

Table 8 illustrates the performance disparity between single-modal signals and the proposed multimodal framework. Following identical preprocessing and feature extraction protocols, EEG consistently exhibits superior discriminative power over PPG, underscoring the heightened sensitivity of cortical neural activity to emotional fluctuations compared to peripheral physiological responses. However, integrating both modalities via SAWGAN-BDCMA yields a significant performance leap. While PPG shows lower standalone accuracy, it contributes vital complementary information from the ANS. These gains validate that the BDCMA mechanism effectively leverages the synergy between central and peripheral signals, capturing emotional nuances inaccessible to any single modality.

**Figure 11:**
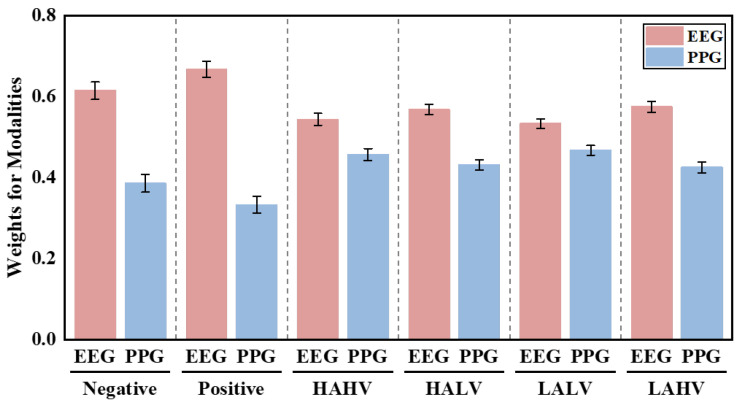
Statistics on the weighting assigned to EEG and PPG signals across different emotion classification tasks on the DEAP dataset.

**Figure 12:**
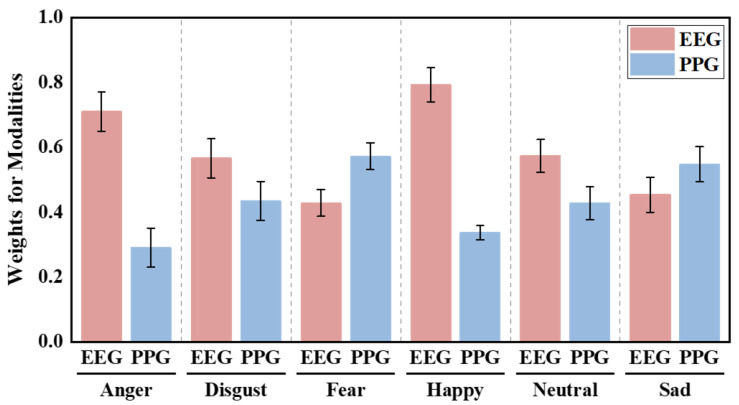
Statistics on the weighting assigned to EEG and PPG signals across different emotion classification tasks on the ECSMP dataset.

Figure 11 and Figure 12 illustrate the average attention weights assigned by the BDCMA model across datasets. On the DEAP dataset, EEG consistently receives higher weights (exceeding 0.6) for valence-related classification, reflecting its direct link to central nervous system activity. While PPG contributions rise in arousal tasks (HAHV and LALV), confirming its efficacy in capturing autonomic cardiovascular dynamics. On the ECSMP dataset, this modality-specific synergy is further nuanced across granular emotion categories. EEG weights peak for `happy’ and `anger’, highlighting distinct cortical patterns in high-intensity states, whereas PPG weighting increases for `fear’ and `sad’, indicating stronger peripheral physiological involvement. These results validate that EEG serves as the primary informational pillar, while PPG provides critical complementary cues, allowing BDCMA to adaptively prioritize the most discriminative modality based on emotional context.

To comprehensively assess the model’s complexity, the parameters of each module were quantified, and both floating point operations per second (Flops) and GPU memory usage were evaluated, as summarized in Table 9. The total number of parameters is approximately 2.23 M, with the SAWGAN generator and discriminator containing 0.71 M and 0.79 M parameters, respectively. The remaining components maintain parameter efficiency while jointly supporting feature extraction and multimodal fusion.

## 5. Conclusions

The SAWGAN-BDCMA multimodal fusion model is developed to overcome insufficient information exchange and suboptimal cross-modal fusion in conventional emotion recognition systems. SAWGAN-based augmentation first increased the balance and diversity of raw EEG and PPG, improving robustness to physiological variability. The dual-branch extractor then captured time-frequency features from EEG and dynamic cardiovascular features from PPG. Finally, the BDCMA module performed deep bidirectional interaction and adaptive fusion, exploiting complementarities between modalities. On the DEAP dataset, SAWGAN-BDCMA achieved 94.25% (binary) and 87.93% (quaternary) accuracy, while on the ECSMP dataset, it reached a peak accuracy of 97.49% for six-class recognition. Furthermore, the analysis of attention weights across emotion tasks confirms the model’s ability to adaptively weigh each modality according to sample properties, which contributes to its robust recognition performance.

Future work will integrate additional physiological channels, such as EMG, GSR and ECG, to further enrich multimodal representations. In parallel, coupling lightweight neural architectures with wearable sensing hardware is expected to enable real-time multimodal emotion recognition, facilitating practical deployment of affective computing in intelligent interaction and health monitoring.

## Figures and Tables

**Table 1:** Principles for assigning labels in the DEAP dataset binary classification task.

Category	Negative	Positive
Label	0	1
Valence Threshold	<5	≥5

**Table 2:** Principles for assigning labels in the DEAP dataset quaternary classification task.

Category *	HAHV	HALV	LALV	LAHV
Label	0	1	2	3
Arousal Threshold	≥5	≥5	<5	<5
Valence Threshold	≥5	<5	<5	≥5

* The quaternary classification categories are defined as: high arousal and high valence (HAHV), high arousal and low valence (HALV), low arousal and low valence (LALV), and low arousal and high valence (LAHV).

**Table 3:** Binary classification performance (%) at different augmentation levels on the DEAP dataset.

Augmentation Level	Accuracy	Precision	Recall	F1-Score
0×	76.01 ± 2.85	76.97 ± 3.26	75.21 ± 3.57	76.09 ± 3.41
0.5×	83.13 ± 1.94	83.51 ± 2.63	82.64 ± 2.79	83.06 ± 2.48
1×	85.17 ± 1.24	84.66 ± 1.17	85.31 ± 1.32	84.96 ± 1.39
2×	94.25 ± 1.36	93.15 ± 1.32	95.14 ± 1.27	94.13 ± 1.52

**Table 4:** Quaternary classification performance (%) at different augmentation levels on the DEAP dataset.

Augmentation Level	Accuracy	Precision	Recall	F1-Score
0×	68.37 ± 3.35	68.35 ± 3.46	68.28 ± 3.52	68.31 ± 3.41
0.5×	71.84 ± 2.17	71.50 ± 1.92	69.40 ± 2.35	70.43 ± 2.09
1×	79.15 ± 1.54	78.60 ± 1.59	78.88 ± 1.67	78.74 ± 1.53
2×	87.93 ± 1.37	87.41 ± 1.32	88.97 ± 1.31	88.18 ± 1.27

**Table 5:** Six-class performance (%) at different enhancement levels on the ECSMP dataset.

Augmentation Level	Accuracy	Precision	Recall	F1-Score
0×	79.75 ± 3.71	79.69 ± 3.63	79.71 ± 3.28	79.68 ± 3.52
0.5×	84.05 ± 1.68	84.01 ± 1.57	84.04 ± 1.65	84.02 ± 1.64
1×	96.25 ± 1.05	96.21 ± 1.03	96.24 ± 0.95	96.20 ± 0.92
2×	97.49 ± 1.16	97.52 ± 1.46	97.51 ± 1.12	97.48 ± 1.38

**Table 6:** Mean accuracy and standard deviation (%) of different methods on the DEAP and ECSMP datasets.

Method	DEAP-Binary	DEAP-Quaternary	ECSMP-Six
SVM	62.53 ± 4.24	59.68 ± 3.42	41.17 ± 1.57
Random Forest	67.95 ± 3.67	64.20 ± 3.84	76.43 ± 3.53
CNN	68.39 ± 2.12	68.43 ± 2.17	74.54 ± 1.82
Husformer [12]	82.67 ± 2.20	78.92 ± 3.22	83.48 ± 2.37
DAIL [11]	84.46 ± 2.74	80.46 ± 3.52	89.36 ± 2.14
Multimodal-Driven Fusion Data Augmentation Framework [24]	92.85 ± 1.04	84.05 ± 1.25	92.75 ± 1.58
MCMT [13]	92.63 ± 3.95	87.36 ± 3.82	93.58 ± 2.74
The proposed model	94.25 ± 1.85	87.93 ± 1.56	97.49 ± 1.64

**Table 7:** Comparison of mean accuracy (%) for emotion recognition across different activation functions within the ViT-CBAM module.

Activation Function	DEAP-Quaternary	ECSMP-Six
ReLU	84.26	95.00
LeakyReLU	84.67	96.25
ELU	86.45	97.50
GELU (Proposed)	87.93	97.49

**Table 8:** Emotion recognition accuracy (%) for single-modal signal and multimodal fusion.

Modal Types	DEAP-Binary	DEAP-Quaternary	ECSMP-Six
EEG	86.36	81.25	90.74
PPG	64.76	62.08	55.03
EEG + PPG	94.25	87.93	97.49

**Table 9:** Analysis of the complexity of the framework.

Module	Params (M)	Flops (M)	Peak GPU Memory (MB)
SAWGAN Generator	0.71	3.83	74.32
SAWGAN Discriminator	0.79	19.11	74.32
ViT-CBAM	0.16	19.58	2.91
TCN	0.17	45.54	9.22
BDCMA	0.40	0.52	1.62

## Data Availability

The original data presented in the study are openly available in the DEAP dataset repository at https://aistudio.baidu.com/datasetdetail/35543 (accessed on 20 July 2024) and the ECSMP dataset at https://data.mendeley.com/datasets/vn5nknh3mn/2 (accessed on 8 November 2025).
